# The biology of jumbo phages

**DOI:** 10.1038/s41467-026-74333-0

**Published:** 2026-06-19

**Authors:** Sam P. B. van Beljouw, Li Yuping

**Affiliations:** Biozentrum, University of Basel, Basel, Switzerland

**Keywords:** Bacteriophages, Phage biology

## Abstract

Jumbo bacteriophages are bacterial viruses with double-stranded DNA genomes exceeding 200 kb. These viral giants feature exceptionally large virions, expansive genetic repertoires, and in some cases, remarkable eukaryotic-like traits. Jumbo phages challenge long-standing notions of phage simplicity, redefining the boundaries of what a phage can be. In this Review, we examine the biology of jumbo bacteriophages, highlighting their diversity, evolutionary origins, distinctive morphologies and lifecycles, complex interactions with bacterial hosts, and their potential for biotechnology and therapy, with a focus on, but not limited to, the *Chimalliviridae* phages.

## Abstract (web summary)

Jumbo phages are bacterial viruses with double-stranded DNA genomes exceeding 200 kb. In this Review, Sam van Beljouw & Li Yuping examine the biology of these bacteriophages, highlighting their diversity, evolutionary origins, distinctive morphologies and lifecycles, interactions with bacterial hosts, and their potential for biotechnology and therapy.

## Introduction

Bacteriophages, or phages for short, are viruses that infect bacteria. They are major contributors to the emergence of biological complexity due to their extensive involvement in horizontal gene transfer (HGT) and the host-parasite arms race^1–3^. Phages are the most abundant biological entities on the planet and are responsible for a sizable fraction of global ecological processes, including material and energy flows in the carbon cycle^4^. Phage research fueled the discovery of foundational principles of molecular biology (e.g., the demonstration that DNA and not protein carries genetic information^5^, the triplet nature of the genetic code^6^, and the elucidation of the operon model^7^) and the development of gene-editing technology (e.g., restriction enzymes^8^ and CRISPR-Cas^9^). Furthermore, being natural enemies of bacteria, phages are promising therapeutic agents to combat bacterial infections^10^.

**Fig. 1:**
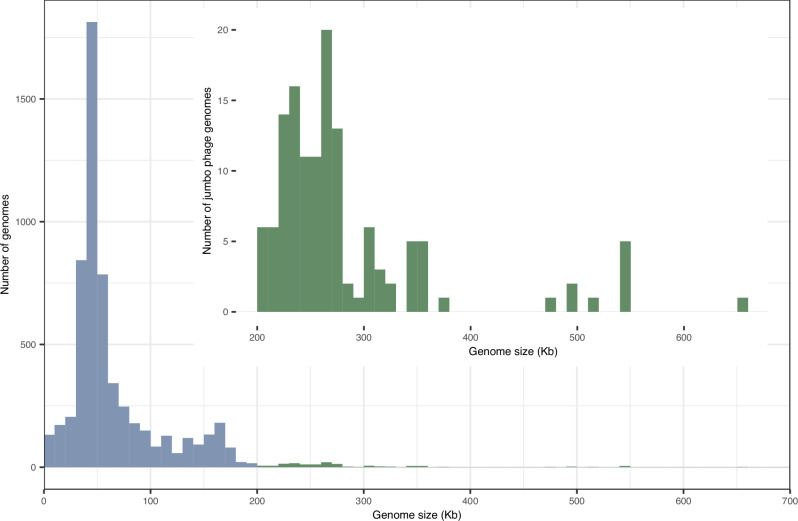
Size distribution of phage genome. Phage genomes <200 kb are plotted in blue and phage genomes >200 kb are plotted in green. The inset zooms in on the size distribution of the jumbo phage genome. The plot was generated using phage genome data from the ICTV 2024 Virus Metadata Resource (VMR; version MSL40.v2), downloaded from the ICTV website. Viruses with Host/Source annotated as archaea or bacteria were extracted as phages. Phages with partial genomes and ssRNA phages assembled from metagenomic datasets were excluded prior to analysis. Genome size information for the remaining phages was retrieved from the NCBI website.

Constant evolutionary pressure on phages' genomes, driven by rapid replication cycles and high genetic turnover, typically minimizes superfluous sequences. Phages therefore generally have compact genomes with a mean size of 52 kilobases (kb)^11^, making the existence of vastly larger genomes all the more striking (Fig. 1). Genomic gigantism in the phage world is exemplified by ‘jumbo phages’, a vaguely defined class characterized by double-stranded DNA genomes exceeding 200 kb^12^. ‘Megaphages’ were originally defined as phages with genomes larger than 540 kb^13^, such as the >540 kb *Prevotella* phages discovered in the human gut microbiome and the >650 kb marine megaphage Mar_Mega_1^14^. However, in practice, megaphages are often referred to as phages with >500 kb genomes. Some of the largest known phage genomes are up to 735 kb^11^, which significantly exceeds the size of some bacterial genomes. Jumbo phages are also notable for their physical dimensions^15^. For example, *Lysinibacillus* phage G measures about 455 nm from head to tail, with a head diameter of about 180 nm^15,16^. In comparison, the dimensions of bacterium *Escherichia coli* are roughly 1 µm by 2 µm^17^, theoretically only fitting a couple of lined-up G phages.

Traditional phage isolation methods bias against the inclusion of jumbo phages. Due to their large virion size, jumbo phages are less likely to pass commonly applied physical filtering steps to separate bacterial cells from phage particles, and diffuse poorly in agar, often failing to establish visible plaques^18,19^. Nevertheless, jumbo phages have been isolated from a wide range of environments, including soil, marine sediments, compost, irrigated fields, chicken feces, tree bark, branches, seawater, blossoms, mudflat, river water, wounds, brine, fracking water, peat, bog, agave, subway wood and deep shale^18,20–22^ infecting bacteria across multiple classes and phyla, including Betaproteobacteria, Alphaproteobacteria, Zetaproteobacteria, Gammaproteobacteria, Cyanobacteria, Bacteroidota, Firmicutes, and Actinobacteria, and notably being able to infect various pathogenic genera such as *Staphylococcus*, *Pseudomonas*, *Salmonella*, *Enterobacter*, *Cronobacter*, *Escherichia*, *Serratia*, *Klebsiella*, *Xanthomonas*, *Dickeya*, *Ralstonia, Burkholderia*^23–31^. Gut-associated jumbo phages have now been reported in diverse animal hosts and in the human intestine, emphasizing that these viruses are members of host-associated microbiomes^13,32,33^. In the global ocean, at least five marine jumbo phage clusters with divergent replication modules and depth-stratified biogeography were found, including nucleus-forming phages enriched in the deep ocean^22^. This all indicates that jumbo phages are abundant, taxonomically and ecologically diverse, occupying a wide host range, even though their representation in culture collections still lags behind their apparent prevalence in nature. The first reported jumbo phages were phage G, isolated in 1968^34^, and *Pseudomonas* phage phiKZ, isolated in 1978^35^. Additionally, phiKZ was among the first jumbo phages to be genomically characterized: its complete 280 kb genome, encoding 371 open reading frames, was sequenced in 2002^20,36^. Subsequent genomic sequencing of other jumbo phages revealed highly mosaic genomes^37^, the majority of which have little, if any, similarity with sequences in the public databases. Even so, almost 50 years of jumbo phage study has revealed a plethora of remarkable aspects of their biology, such as self-encoded multi-subunit RNA polymerases (RNAPs) and various eukaryotic-like features.

In this review, we explore the biology of jumbo bacteriophages, beginning with their diversity and evolutionary history, examining key features of the jumbo phage lifecycle with a focus on the well-studied *Chimalliviridae* phages, and discussing the unique structural characteristics found in some jumbo phages. We further summarize current insights of bacterial immunity antagonizing jumbo phages and the counteraction by these viruses. Finally, we consider their biotechnological and therapeutic potential.

### Evolution and classification

#### Jumbo phage evolution

Beyond the historical, somewhat arbitrary 200 kb genome size cutoff for jumbo phage categorization, an analysis of phage genome and proteome size distribution determined that genomes larger than 180 kb define ‘jumbo’ phages^23^. Phylogenetic analysis suggests that jumbo phages originated multiple times independently from smaller tailed phages, with at least 19 separate emergences^38^. Many jumbo phages are typified by proteins also found in various smaller phages^23^. For example, some jumbo phages can be clustered based on the presence of coliphage T4 proteins and can thus be seen as evolutionary descendants from a T4-like phage^23^. Moreover, the display of both myovirus-like and siphovirus-like morphologies hints that jumbo phages are not an evolutionary unitary group, evolving instead on separate occasions^23^. Interestingly, jumbo phages possess the myovirus-like morphology more often than siphovirus-like morphology, and no cases have been reported of jumbo phages evolving from podovirus-like lineages, alluding to pre-adaptations in myovirus-like phages favoring the evolution of jumbo phages^23^. Furthermore, proteins in jumbo phages tend to be significantly longer than those in smaller phages, suggesting a correlation between protein size and genome size^23^.

The emergence of jumbo phages was speculated to be driven by mutations in capsid proteins, which allowed for an increased capsid volume, subsequently accommodating a larger genome and more genes^39^. The increased genome size could be derived from the duplication of existing genes or horizontal gene transfer (HGT) caused by mobile genetic elements (MGEs). For example, some jumbo phages harbor genomic arrays of more than 30 highly similar genes, suggesting genome expansion through duplication^39^. Additionally, signs of MGEs have been observed in many jumbo phage genomes, such as homing endonucleases (see below), introns^40,41^, self-splicing introns^23,42^, inteins^23^, tyrosine integrase dependent mobile DNA^43^, with a general mosaic gene architecture potentially caused by MGE activity^37^.

Although HGT is an obvious driver of biological innovation, limited gene flow and genetic exchange between lineages are needed for speciation. Some jumbo phages deploy intriguing strategies to achieve this, termed ‘subcellular genetic isolation’ and ‘virogenesis incompatibility’^44,45^. Subcellular genetic isolation arises through the early phage infection (EPI) vesicle and the nuclear shell during infection of *Chimalliviridae* phages (see “Life cycle”), spatially separating genomes to limit recombination. Further reproductive isolation arises from virogenesis incompatibility, whereby viable progeny production is limited during co-infection with different viruses. For example, PhuZ monomers (needed for nucleus positioning; see “Life cycle”) of closely-related phiPA3 and phiKZ phages co-assemble into non-functional hybrid filaments during co-infection, limiting progeny production^44^. This creates selective pressure against co-infection, promoting lineage-specific evolution. Another example of a phage speciation factor is the homing endonuclease, which recognizes and nicks the homologous sequence in the polymerase gene of the competitor phage to block reproduction of the rival^42,44^, driving divergence of these viral lineages. Other phage exclusion mechanisms may be encoded by Ter systems found on jumbo phage genomes^23^. Ter systems encode nucleic acid sensors and components that may change the membrane, suggesting that jumbo phages could sense a competing virus and prevent its entry. Moreover, some jumbo phages encode gene clusters for lipopolysaccharide production, potentially to decorate the bacterial membrane with a surface unrecognizable by competitor phages^23^.

#### Jumbo phage classification

Genome analysis showed no universally conserved genes at the sequence level across all jumbo phages; instead, comparative genomics of jumbo phages identified seven gene clusters conserved in over half of the genomes, including the nuclease SbcCD subunit (DNA double-strand break processing), ribonucleotide reductase beta subunit (deoxyribonucleotide synthesis), DNA primase-helicase (DNA unwinding and primer synthesis), sliding clamp loader subunit (processive DNA replication), a hypothetical protein (unknown function), thymidylate synthase (dTMP biosynthesis), and dihydrofolate reductase (folate recycling for nucleotide synthesis)^37^. Jumbo phages also encode numerous homologs of cellular recombination and repair proteins (e.g., Holliday junction resolvases, recombinases, topoisomerases, endonucleases), suggesting their reliance on mechanisms such as recombination-dependent replication, non-homologous end joining, and genome topology management^23^. Hundreds of jumbo phages encode chimallin, the main component of the phage nucleus (see “Life cycle”)^21,46^. These phages form a monophyletic group, termed ‘*Chimalliviridae*’, which may have arisen from a common ancestor. *Chimalliviridae* are predicted to infect a wide range of host bacteria from diverse environments, including plants and humans^21,46,47^. Members from the *Chimalliviridae* family contain about 70 conserved genes, including chimallin, the major capsid protein, the large terminase subunit, RNAP subunits, and 53 genes of unknown function^21^.

Although *Chimalliviridae* are currently the best-characterized jumbo phages, comparative genomics and environmental surveys show that they represent only a subset of jumbo phage diversity^22,23,37,38^. Many of the jumbo phages lack chimallin homologs and are thus unlikely to form a proteinaceous phage nucleus as the *Chimalliviridae*. Outside of *Chimalliviridae*, jumbo phages such as phage G, moraphages, AR9-like phages, and *Pseudomonas* phage MIJ3 contain expanded repertoires of tRNAs, translation-related factors, unusual metabolic pathways, or hypermodified DNA^23,26,48,49^; and *E. coli* jumbo phage Sharanji induces cell filamentation^31^. Collectively, this alludes to the vast diversity of jumbo phage genetics and infection strategies despite the so far limited studies of jumbo phages.

### Life cycle

**Fig. 2:**
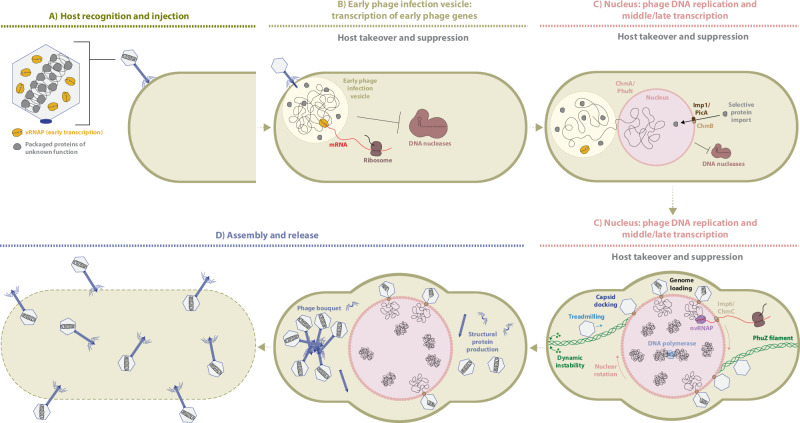
Key stages of the *Chimalliviridae* lifecycle. **A** *Chimalliviridae* phages recognize the cell surface of their host. The genome of some jumbo phages is packaged in the capsid together with various proteins, including several copies of the virion RNA polymerase (vRNAP). **B** Upon injection into the cell, an EPI vesicle is formed, within which vRNAP injected along with phage genome drive transcription of the early phage genes. The EPI vesicle shields the injected DNA from DNA nucleases. **C** Upon formation of the nucleus, there is likely a hand-off of the genome from the EPI vesicle to the nucleus. The nucleus grows over time by the addition of ChmA/PhuN proteins and is pushed to mid-cell by the dynamic instability of the PhuZ filaments. The phage genome is replicated inside the nucleus and loaded via ChmB pores into the capsids, which dock onto the rotating nucleus after being transported there via ‘treadmilling’ over the PhuZ filaments. Transcription inside the nucleus is performed by the non-virion RNA polymerase (nvRNAP), and mRNA molecules are exported into the cytoplasm, potentially with the help of Imp6/ChmC. Protein import complexes composed of, amongst others, Imp1/PicA, selectively import proteins into the nucleus. Similar to the EPI vesicle, the nucleus physically prevents the access of DNA nucleases from phage genomes. **D** Structural proteins are produced in the cytoplasm and assembled into progeny phage particles, which, for some jumbo phages, further assemble into phage bouquets. In the end, mature phages are released into the environment by host lysis.

**Fig. 3:**
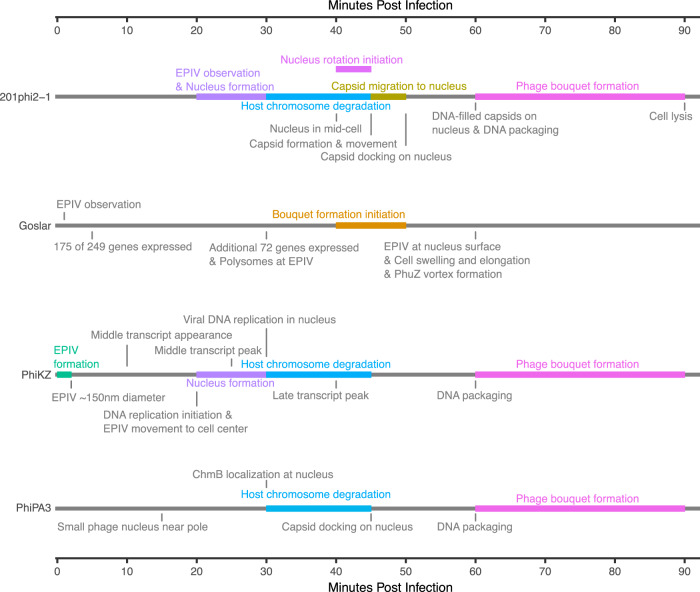
Timeline of key stages in the lifecycle of *Chimalliviridae* phages. The timepoints shown are compiled from multiple independent studies. Exact timings of individual events may vary between studies due to differences in experimental conditions, methodologies, and interpretations of the same lifecycle stages. Nevertheless, when considered together, they provide a representative temporal overview of the lifecycles of *Chimalliviridae* phages 201phi2-1^66,91,92,103,117^, Goslar^66,69,95^, phiKZ^67,68,78,117,120^, and phiPA3^91,108,117,120^. EPIV early phage infection vesicle.

Many jumbo phages have uniquely complex lifestyles with intricate viral organization. The lifecycles of the *Chimalliviridae* phages^50–52^ involve two compartments (the EPI vesicle and a nucleus-like structure, called the ‘pseudo-nucleus’ or simply ‘nucleus’), cytoskeletal filaments, and entirely host-independent transcription (Figs. 2 and 3). Here, we go through the different stages of the *Chimalliviridae* lifecycle, from infection to release, as well as providing features of lesser studied jumbo phages.

#### Host recognition and injection

A typical phage lifecycle begins with interaction between phage tail fiber proteins and host cellular elements, which for some involves the type IV pilus, flagella, and/or intact LPS (Fig. 2A)^53–58^. Successful interaction leads to the active injection of the viral genome into the host cell, sometimes accompanied by co-injected proteins that jumpstart viral life inside the cell^59^.  It has been shown that in addition to its structural proteins, phiKZ packages several head proteins, including the subunits of the virion RNA polymerase (vRNAP), a protease, a predicted helicase, and many other proteins with unknown function. Out of which six abundant head proteins, known as the inner body (IB) proteins (gp89, gp90, gp93, gp95, gp97, and gp162), are each present at 100–200 copies within the virion. Many of the head proteins, such as vRNAP and some IB proteins, are injected into the host along with phiKZ genome^60–62^. Interestingly, the genes encoding five of these six IB proteins are in the same gene cluster, despite overall low synteny in the phiKZ genome^60,62^. However, this apparent clustering should not be interpreted as a general rule for the organization of co-functional jumbo phage proteins, as phiKZ proteins encoded as far as >100 kb apart could still be interaction partners, such as the subunits of the RNA polymerase^62^.

Despite their large genomes and particles, some jumbo phages can adsorb quickly and complete lytic cycles on time scales comparable to, or slightly longer than, those of smaller Caudoviricetes. For example, *Vibrio* jumbo phage vB_VhaM_pir03 reaches ~90% of irreversible adsorption within 6 min and has a ~40 min latent period, and *Staphylococcus* phage SA1 adsorbs almost completely within 5 min and has a latent period of ~55 min^63,64^. Considerably longer latent periods have also been reported for some jumbo phages, with *Erwinia* phage Deimos-Minion needing 3–4 h to complete its life cycle^65^.

#### Early phage infection vesicle: transcription of early phage genes

Early phage infection (EPI) vesicles (previously known as unidentified spherical bodies^66^ and round compartments^67^) are membrane-derived compartments observed during early stages of *Chimalliviridae* phage infection^59,66–69^ (Figs. 2B and 3). They are spherical and vary in size, typically with a membrane thickness of 4–5 nm and measuring 150–260 nm in diameter, depending on the phage and stage of infection^66,68,69^. EPI vesicles possess a volume 3–4 times that of the phage capsid, sufficient to accommodate the injected DNA and associated proteins^66,69^. The vesicles appear to be derived from the host inner membrane^68^ and spatially segregate phage and host machineries, shielding phage DNA from host nucleases and preventing transcription of host genes by injected viral polymerases^59,70^ (Fig. 2A). Cryo-electron tomography showed the inner tube of the phiKZ virion attached to the EPI vesicle during DNA injection, suggesting a direct role of the inner tube in EPI vesicle formation with perhaps DNA injection inflating the EPI vesicle^68^. The EPI vesicle is a transient compartment for essential phage processes such as transcription of early genes^69,71^, mirroring mechanisms seen in eukaryotic viruses^72^.

Upon injection into the EPI vesicle, phage genome transcription starts immediately^59,71,73^. Host-independent transcription was demonstrated by the observation that infection was unaffected by the host RNAP inhibitor rifampicin^74–76^. PhiKZ and related phages encode two sets of RNAPs: the virion RNAP or vRNAP, which is injected into the EPI vesicle and responsible for early transcription, and the non-virion RNAP or nvRNAP that is synthesized de novo post phage infection. In phiKZ, upon injection, the vRNAPs, possibly with additional co-injected proteins (e.g., IB proteins)^21,59,62,77^, initiate transcription from 28 early promoters to drive gene expression^78^, including the expression of nvRNAP subunits^70,78,79^. The nvRNAP is localized in the later assembled nucleus-like compartment of phiKZ (Fig. 2C), whereas the vRNAP is excluded from the nucleus, indicating that compartmentalization aids spatial and temporal coordination of transcription^71^. For jumbo phages that do not form a nucleus, one strategy employed to avoid host DNA transcription is the use of base uracil instead of thymine in the phage genome, promoting transcription initiation by viral RNAPs^80^.

RNA expression profiling has shown that phiKZ entirely relies on its own multi-subunit transcription machinery^78^. In contrast, most known small phages require host transcription machinery at some point during their lifecycle, and, if encoded, small phage RNAPs are single-subunit instead of multi-subunit (e.g., T7^81^, N4^82^, and SP6^83^). The jumbo phage RNAPs are evolutionarily related to bacterial RNAPs, albeit showing notable differences^40^. The bacterial RNAP is composed of the main subunits β and β′, stabilized by a dimer of α subunits and a ω subunit, with promoter specificity defined by the σ subunit^84^. The core of jumbo phage RNAP is similarly composed of multiple β/β′-like subunits^60,78,79^. Specifically, the vRNAP core comprises at least five polypeptides corresponding to the full-length β′ and β subunits of bacterial RNAP, while the nvRNAP core consists of at least four such polypeptides^70^. It remains unknown whether the vRNAP and nvRNAP emerged from gene duplication in the ancestral jumbo phage genome or from two distinct acquisition events. Notably different from bacterial RNAPs is the absence of homologs of the bacterial α, σ, or ω subunits, and phage RNAPs do not stably interact with these bacterial subunits, pointing to other means for phage RNAP assembly, stability and transcription initiation^40,70,79^. For the nvRNAP of phiKZ, a subunit was identified, namely GP68, that has structural homology to bacterial σ factors and is essential for promoter recognition, suggesting similarity in function^85,86^. In *Bacillus* jumbo phage AR9, which is not a member of the *Chimalliviridae* family, a fifth protein (GP226) was found to be contained in the nvRNAP, which, although presenting no sequence similarity to σ, is essential for transcription initiation^80^. Moreover, some RNAP-containing jumbo phages encode homologs of proteins involved in the takeover of host transcriptional apparatus, such as ADP-ribosyltransferases^87^, GCN5-like NH2-group acetyltransferases^88^, and anti-sigma factors^89^, suggesting that they can interface with host transcription despite their self-sufficiency in transcription^23^.

Only a small subset of jumbo phages, most notably members of the *Chimalliviridae* and *Straboviridae* families, encode multisubunit RNAPs, whereas the majority of jumbo phages lack these enzymes and instead depend on the host RNAP for transcription^38,70^. These RNAP-lacking jumbo phages often encode well-conserved homologs of bacterial σ factors, which likely associate with the host RNAP to initiate transcription from phage-specific promoters^70^. Evolutionarily, jumbo phage RNAP subunits present unique combinations of insertions and fusions, unparalleled in their cellular counterparts^23^. This raised the suggestion that instead of being derived from cellular enzymes, viral RNAPs are remnants of transcription machinery in the primordial replicator pool, predating the last universal common ancestor and potentially contributing to cellular RNAP evolution^23,90^.

#### Nucleus: phage DNA replication and middle/late transcription

The remarkable ability of *Chimalliviridae* phages to construct a nucleus-like compartment for replication has been experimentally demonstrated for *Pseudomonas* phages phiKZ^67,91^, 201phi2-1^92^, and phiPA3^91,93^, *Serratia* phage PCH45^94^, *E. coli* phage Goslar^66,95^, *Erwinia* phages RAY^21^ and Asesino^96^, *Vibrio* phages Ariel and Eric^97^, and *Salmonella* phage SPN3US^98^ (Fig. 2C). Similar to eukaryotic cells, nuclei of jumbo phages act as genome replication factories, separate DNA replication and transcription from translation, and facilitate regulated trafficking of nucleic acids and proteins.

Phage nucleus formation starts about 20 min post-infection (MPI) (Fig. 3) and is initiated by chimallin protein expression, encoded by the *ChmA* gene (named after the Chīmalli shield used by indigenous Mesoamerican warriors), also known as ‘phage nucleus enclosure protein’ (PhuN), which is one of the first jumbo phage genes transcribed and produced in high abundance^66,92,93^. ChmA self-assembles into a flexible, proteinaceous shell with a thickness of about 5–6 nm, which encloses phage DNA and grows larger in diameter than the width of the cell during phage DNA replication^66,92^. Enzymes involved in DNA replication and transcription (e.g., DNA helicase, ligase, RNase, RecA, nvRNAP subunits and host-derived topoisomerase I) are synthesized in the bacterial cytoplasm and imported inside the nucleus, while host translation factors and nucleases remain excluded^92,99,100^. This leads to complete resistance of nucleus-encoding phages to known DNA-targeting immune systems, including restriction enzymes and CRISPR-Cas effectors^99–101^ (see Bacterial defense against jumbo phages and jumbo phage anti-defense).

The nucleus structure consists of negatively charged repeating tetramers of 11.5 × 11.5 nm that likely repel DNA^66^, facilitating efficient compartmental replication and transcription. Lattice pore sizes range from fully closed to 2.3 nm, conferring semi-permeability that likely allows passage of small molecules (e.g., metabolites, nucleotides, amino acids) and RNA transcripts, but not folded proteins^66,102^. Since ChmA subunits do not spontaneously assemble into a nucleus in uninfected cells, it likely requires additional phage components to promote assembly^92^. EPI vesicles are often observed physically connected to the developing phage nucleus, rotating synchronously, potentially facilitating genome transfer via an unknown portal mechanism^69^. This spatial coupling points to nucleus assembly adjacent to, or on the surface of, the EPI vesicle.

The nucleus is positioned in the center of the cell by the phage-encoded tubulin-like protein PhuZ^103^. PhuZ forms dynamic, triple-stranded (e.g., for phiKZ) or five-stranded (e.g., for Ray) bipolar spindles that guide the nucleus via treadmilling, in which subunits are added at the minus-end (near the poles) and removed at the plus-end (oriented toward mid-cell)^21,45,103–107^. The efficiency of PhuZ-coordinated nucleus centering is dependent on host cell length^107^. Progeny capsids, assembled on the host membrane, traffic along PhuZ filaments toward the nucleus at ~50 nm/s, where they dock onto pores formed by ChmB in the ChmA shell for genome loading^21,95,103,108,109^.

The forces generated by the growing PhuZ filaments at both sides of the nucleus induce rotation, which evenly distributes capsids over the nucleus surface^92^. Although non-essential, PhuZ increases replication efficiency by ~50% for some *Chimalliviridae* phages^77,104^. Without PhuZ filaments, nuclei localize to the poles, leading to reduced phage yield, likely by reduced surface availability for capsid docking^96,104^. Interestingly, Goslar does not position the nucleus at mid-cell, seemingly separating the nucleus and bacterial inner membrane through its unique vortex-like cytoskeletal structure that wraps around the nucleus while connecting radially at the membrane, driving nucleus rotation through dynamic instability^95^. Many chimallin-encoding phages do not carry PhuZ homologs, further emphasizing their non-essentiality for replication^21^. For example, Ariel and Asesino do not deploy PhuZ filaments^96,97^. Instead, Ariel infection causes the host cell to become spheroidal, alleviating the need for mid-cell positioning because the poles are lost^97^, whereas the nucleus of Asesino is physically positioned mid-cell by two flanking bacterial nucleoids^96^.

Phage mRNA export from nucleus to the cytoplasm for translation may occur via ChmA nuclear pores and accessory proteins such as ChmC/Imp6, a nucleic acid-binding factor with high affinity for phage mRNA and small regulatory RNAs^109^. Given the close genomic localization of *ChmC/Imp6* with RNAP genes, ChmC might be physically associated with the transcription machinery^109^. Translocated mRNA molecules of phiKZ strongly co-localize with host ribosomes, where they are potentially prioritized for translation over host transcripts^110^. Additionally, ChmB may also contribute to RNA and protein trafficking across the nucleus^108,109^. PhiKZ was also found to express non-coding RNAs, suggesting potential roles in regulating phage gene expression or modulating host interactions^110^.

Jumbo phages have evolved a selective, sophisticated system for importing proteins into their nucleus-like compartment. Surprisingly, a specific GFP mutant was also transported into the nucleus, suggesting that cargo entry is dictated by intrinsic features of the cargo protein^101^. Recent studies found that the import pathway is mediated by a set of specialized factors, with Imp1/PicA playing a central role^111,112^. Imp1/PicA localizes to the nucleus shell and is almost universally co-encoded with ChmA, suggesting a conserved role in phage nucleus function^111,112^. Moreover, Imp3 is required for Imp1 function, and additional proteins Imp4, Imp5, and ChmC/Imp6 contribute in various combinations depending on the cargo. Nuclear import does not rely on conventional terminal signals; rather, surface recognition motifs on cargo proteins determine recognition and trafficking. Additionally, various residues on the Imp1/PicA surface are critical for cargo selection, indicating its role in selective import^111,112^. This explains the high selectivity of the import pathway and its ability to exclude toxic elements (e.g., bacterial defense proteins or products from co-infecting phages).

#### Host takeover and suppression

PhiKZ infection profoundly impacts host cellular processes, particularly by altering host transcript levels and remodeling host protein complexes (Fig. 2B, C). Transcriptomics revealed that phiKZ imposes strong transcriptional control over the host, modulating host gene expression within minutes of infection^110^. Affected host transcripts included those involved in cellular energy production, which were found to disassociate from ribosomes during infection^110^. Phage transcripts account for around 45% of all transcripts at 10 MPI, which increases to around 70% at 35 MPI^73,78,113^. mRNAs encoding phage proteins that interact with ribosomes accumulate faster than other early phiKZ transcripts (i.e., nvRNAP subunits, ChmA and PhuZ), suggesting host ribosome modulation occurs very early on^73^. Moreover, numerous phiKZ proteins have been found to interact with bacterial translation machinery components, including 70S ribosomes, ribosomal stalk proteins, ribosome silencing factors and proteins involved in polypeptide folding^62^. The tRNAs and RNA ligases found in jumbo phages^23,114^ further suggest phage interference with host translation, potentially via preferential phage protein translation, or preventing/reversing tRNA cleavage by host RNases^115^. Concurrently, phiKZ expresses proteins that interact with bacterial RNA degradation machinery to shield viral RNAs^116^.

The fate of the bacterial chromosome depends on the infecting jumbo phage. During phiKZ infection, the bacterial genome was found degraded at 30 MPI^117^ (Fig. 3) or diffuse in the cytoplasm^113^, possibly by the action of stress-induced protective DNA-binding proteins^67^. Host genomes were not degraded by RAY and Asesino^21,96^. RAY even expresses a transcriptional regulator that binds the host genome, potentially to steer bacterial transcription^21^. Conversely, bacterial genome completely degrades during infection with 201phi1-2 and Goslar, perhaps to free nucleotides for phage genome replication and provide space for nucleus growth^95,105,117^. Changes to host cell shape also differ among infecting phages: infection by phiKZ, Goslar, Ariel and Eric cause significant bulging of the cell envelope^67,91,92,95,97^, whereas RAY and PCH45 have little effect on cell shape^21,94^.

Given the wide coding potential of jumbo phage genomes, complementing the infected host with gene products necessary for phage propagation is likely a common theme. For example, cyanobacteria-infecting jumbo phages encode genes involved in photosynthesis and carbon metabolism, perhaps helping maintain photosynthesis and energy production during infection^118^. Moreover, some jumbo phages encode enzymes needed for synthesizing NAD+, likely ensuring the resource supply needed for rapid DNA synthesis^32,119^.

#### Assembly and release

The last step in a lytic phage lifecycle is release of virion particles (Fig. 2D). A structural arrangement known as the ‘phage bouquet’, named due to its similarity to a floral bouquet^117^, has been observed in the cytoplasm during the final stages of the infection cycle of Goslar, phiPA3, phiKZ and 201phi2-1^95,117^ (Fig. 3). Here, mature virions form spherical clusters, with tails pointing inward and capsids radiating outward. Because of their dense packing, bouquets contain only minimal amounts of cytoplasmic components such as ribosomes, implying a distinct internal physiology^117^. Over time, bouquets increase in size as virions are incorporated^117^; they may even exceed the size of the phage nucleus, reaching dimensions of ~2 μm by 5 μm for Goslar phages^95^. This surpasses the size of an uninfected cell, as the host cell itself enlarges substantially during infection. Phage bouquets are not observed during RAY infection, suggesting they are not essential for virion production^21,117^.

Various burst sizes have been measured for jumbo phages, including ~39 for phiKZ^77^, ~117 for SPN3US^120^, ~80 for AR9^40^, and ~165 for *Pseudomonas* phage AttikonH10^121^. Also, markedly smaller burst sizes were measured for some jumbo phages, such as ~5 for Deimos-Minion^65^ and ~7 for *Pseudomonas* phage KTN4^53^. Interestingly, genes of the filamentous prophage Pf1 were upregulated during phiKZ infection, possibly because the prophage tries to escape the dying cell^78^.

### Structural features

**Fig. 4:**
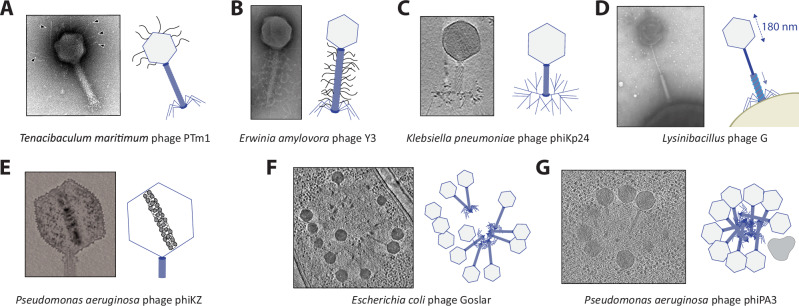
Examples of jumbo phage morphology and structure. **A** *Tenacibaculum maritimum* phage PTm1 displays hair-like protrusions from the capsid. This figure panel^130^ is reprinted with permission. **B** The tail sheath of *Erwinia amylovora* phage Y3 is decorated with hair-like appendages. This figure panel is reproduced from^131^. Copyright © 2026. Elsevier Masson SAS. All rights reserved. **C** *Klebsiella pneumoniae* phage phiKp24 possesses a hyperbranched tail fiber structure. This figure panel^127^ is licensed under CC BY 4.0 (https://creativecommons.org/licenses/by/4.0/). **D** *Lysinibacillus* phage G has the largest known capsid with a diameter of 180 nm and has its tail sheath detached from the head upon injection. This figure panel^16^ is reprinted with permission. **E** The capsid of *Pseudomonas aeruginosa* phage phiKZ contains a cylindrical structure made from proteins and DNA. This figure panel^61^ is reprinted with permission. (**F**) *Escherichia coli* phage Goslar virions form phage bouquets with capsids localized at the center, whereas (**G**) *Pseudomonas aeruginosa* phage phiPA3 phage bouquets are horseshoe-shaped with an unknown structure at the entrance. The figure panel^95^ in (**F**) is reprinted with permission. The figure panel^117^ in (**G**) is licensed under CC BY 4.0 (https://creativecommons.org/licenses/by/4.0/).

Jumbo phages show a range of exotic morphologies and structural features (Fig. 4). A hallmark is their large capsids, such as the ~145 nm head of phiKZ^122–124^, ~142 nm head of RAY^21^, and the ~180 nm head of phage G^16^. Cryo-electron microscopy studies have shown that icosahedral geometry is common among jumbo phage capsids^16,39,122–127^.

PhiKZ incorporates at least 40 distinct structural proteins^128,129^. It has a 200 nm long tail with an inner diameter of 4.5 nm and an outer diameter of 11 nm, along with a baseplate measuring 80 nm across and 35 nm in thickness, with tail fibers extending ~50 nm^122,123^. Mass spectrometry studies have identified about 30 proteins associated with the phiKZ head, of which only around 10 are structural^60,124,128^. Its head is composed of a major capsid protein and roughly ten minor capsid proteins. Two of these minor proteins decorate the outer surface of the capsid, while the others are attached to its inner structure. While empty phiKZ capsids measure ~85 nm in diameter, DNA-filled ones reach about ~100 nm^67^; this is still smaller than the mature phiKZ capsid (~145 nm), suggesting that capsid maturation continues post-DNA packaging. The maturation of the phiKZ^60^ and SPN3US^120^ heads involve extensive proteolytic processing. In phiKZ, this is carried out by a virally encoded serine protease which recognizes a S/A/G-X-E motif^60^.

Some jumbo phages show particularly exotic head and tail structures. For example, phages PTm1 and PTm5 have capsids decorated with tail fibers, which could enable adsorption to specific bacterial hosts (Fig. 4A)^130^. *Erwinia* phage Y3 features hair-like appendages along its tail (Fig. 4B)^131^, and *Klebsiella* phage phiKp24 possesses a branched tail fiber structure to enable infection of a broad panel of *Klebsiella pneumoniae c*apsular polysaccharide types (Fig. 4C)^127^. Phage G, the largest known laboratory-propagated phage, contains a coiled outer structure wrapped around its tail sheath and an unusual contraction mechanism decoupled from its neck region (Fig. 4D)^132^.

A unique architectural feature of some jumbo phages is the 15–20 MDa IB^60^, a cylindrical proteinaceous scaffold located within the capsid. The IB structure was first observed around ruptured capsids^133^, and is likely playing a central role in genome packaging (Fig. 4E). Electron microscopy showed that the IB of phiKZ is highly ordered, measuring approximately 24 nm in diameter and 105 nm in length, attached at the ends to the inside of the capsid and oriented at a tilt relative to the portal axis^61^. This orientation roughly aligns with the spool-like arrangement of DNA observed in phiKZ capsids, suggesting that the IB serves as the scaffold around which the genome is tightly wound^61,122^. Notably, IB assembly appears to occur before genome packaging, as it is present in nascent capsids in host cytoplasm before DNA loading occurs^67,134^, supporting a model in which the IB guides DNA packaging. IB-like architectures have also been reported in other jumbo phages^98,135–137^, indicating that they may be common to large bacteriophages.

During virion assembly, phage bouquets have been observed with co-localizing free capsids (Goslar)^95^ (Fig. 4F) as well as horseshoe-shaped structures lacking internal capsids entirely but with an unknown dense material at the opening (phiPA3 and, at a reduced frequency, phiKZ and 201phi2-1)^117^ (Fig. 4G). The precise function of bouquets remains unclear, but proposed roles include shielding mature virions from host proteases or enhancing phage maturation^117^.

### Bacterial defense against jumbo phages and jumbo phage anti-defense

**Fig. 5:**
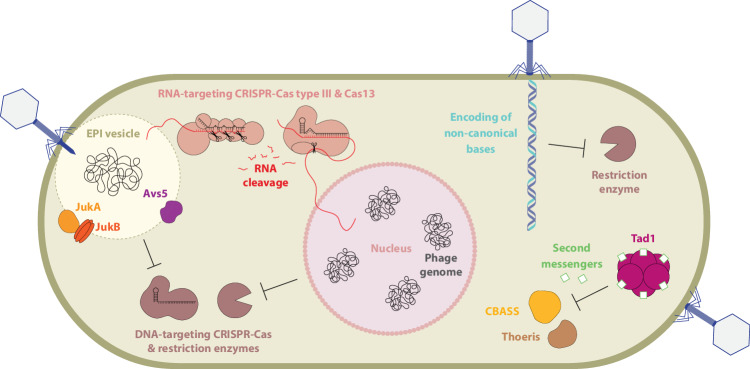
Overview of characterized and predicted defense systems against jumbo phages and counter-defense strategies associated with jumbo phages. The early phage infection (EPI) vesicle and nucleus protect the phage genome from nucleases, such as DNA-targeting CRISPR-Cas and restriction enzymes. RNA-degrading CRISPR-Cas type III and Cas13 can still target the nucleus-forming phages as they have access to phage transcripts. Jumbo phage killer (Juk) proteins JukA and JukB as well as AVAST type 5 (Avs5) interfere with the EPI vesicle to hamper infection. Various jumbo phages encode uracil instead of thymine in their genome, likely preventing targeting by restriction enzymes. PhiKZ encodes Tad1, a protein that counters cyclic oligonucleotide-based antiphage signaling systems (CBASS) and Thoeris immunity through sequestration of cyclic nucleotide second messengers.

The ongoing arms race between MGEs and their bacterial hosts has resulted in the emergence of diverse defense and counter-defense strategies^138,139^. For jumbo phages and their hosts, several of these systems have been experimentally described (Fig. 5), while others are predicted by genomic and structural analyses.

One elegant way *Chimalliviridae* phages counter DNA-targeting defense is via the nucleus, which physically prevents restriction enzymes and DNA-degrading CRISPR-Cas systems from accessing phage genomes^99,100^. However, since jumbo phage transcripts are exported into the cytoplasm for translation, RNA-targeting systems remain effective. For example, type III CRISPR-Cas immunity of *Serratia* antagonizes phage PCH45 via RNA-guided activation of the broad NucC nuclease^140^, which triggers host cell death and curbs phage propagation. Interestingly, strains harboring CRISPR-Cas remain susceptible to jumbo phages lacking ChmA and anti-CRISPR genes, suggesting additional mechanisms of CRISPR-Cas system evasion^37^.

Host bacteria frequently employ nucleotide-derived second messengers to activate defense responses, such as in type III CRISPR-Cas^141^, cyclic oligonucleotide-based antiphage signaling systems (CBASS)^142^, and Thoeris^143^. By encoding enzymes such as phosphodiesterases and NAD+-metabolizing proteins, jumbo phages potentially disrupt these signaling cascades^23^. Moreover, the Tad1 protein, injected into host cells by phiKZ, blocks CBASS and Thoeris immunity by sequestering cyclic nucleotide second messengers^144^. So far, Tad1 is the only anti-defense protein described in jumbo phages^139^. However, given the abundance of small, uncharacterized open reading frames in jumbo phage genomes, it is plausible that a rich arsenal of anti-defense proteins remains to be discovered. Indeed, it was found that anti-defense systems were enriched in jumbo phages compared to smaller phages, suggesting that the acquisition of these systems may have contributed to the evolution of genome gigantism^38^. Some jumbo phages chemically modify their DNA to avoid immune recognition. The incorporation of uracil instead of thymine^40,145–147^ and predicted capabilities for the incorporation of deazaguanine bases in place of guanine and transfer of an acyl group to adenine^23,40^ likely serve to evade host restriction enzymes. Notably, DNA modification systems are less common in *Chimalliviridae* phages, suggesting that phage nuclei may mitigate the need for genomic camouflage. Moreover, methylases encoded by some jumbo phages may act on phage DNA or RNA, possibly shielding their nucleic acids from immune systems^23^.

Unsurprisingly, bacteria have evolved multiple immune strategies to specifically antagonize the unique biology of *Chimilliviridae* phages, among which are the jumbo phage killer (Juk) immune system, AVAST Type 5 (Avs5), Dionysus and Ophion. Juk consists of a sensor protein, JukA, which detects early phage proteins anchored in the EPI vesicle, and a diverse set of effector proteins, including phospholipases and transcriptional regulators^54^. Among the effector proteins is JukB, which is recruited to the infection site by JukA and forms tetrameric pores on the EPI vesicle, presumably disrupting its integrity and facilitating phage DNA degradation by host nucleases. In some instances, a JukA protein alone confers protection though its mode of action remains unclear. JukA is present in over 20% of gram-negative bacteria and is the third most abundant immune protein after restriction-modification and CRISPR-Cas proteins. Avs5 in *P. aeruginosa* consists of a Sir2 NADase that was found to localize to the EPI vesicle, where it recognizes jumbo phage Avs5 defense activator (JADA), an early-expressed protein of unknown function^148^. Binding to JADA activates the NAD+ hydrolysis activity of Avs5, causing phage nucleus disruption and slowing down phage propagation via unknown processes. Interestingly, NAD+ levels have been shown to restore to basal levels at 15 MPI, potentially via NAD+ recycling proteins. Dionysus and Ophion, also found in *P. aeruginosa,* prevent EPI vesicle formation and nucleus formation, respectively^149^. Ophion strongly co-occurs with the Juk system, suggesting various bacterial defense mechanisms targeting different stages of the life cycle of *Chimalliviridae* phages.

### Jumbo phage engineering

Phage engineering has rapidly advanced^150^, but traditional genetic tools often struggle with nucleus-forming phages due to the physical separation of DNA within the protective phage nucleus. However, RNA transcripts exported from the nucleus can be targeted by tools like the CRISPR-Cas RNase Cas13^141^. For instance, catalytically inactive Cas13 variants have been used in CRISPR interference through antisense RNA targeting (CRISPRi-ART)^151^, which suppresses gene expression at the transcript level by binding mRNA. CRISPRi-ART was successfully used to block ChmA production and thus phage nucleus formation^69^.

Furthermore, phage genome editing using homologous recombination has been achieved with a Cas13-based counterselection system, in which only recombinant phages acquiring an anti-Cas13 gene can evade transcript-targeted degradation^77^. A non-genetic method for systematic silencing of phage genes was developed for phiKZ, in which synthetic antisense oligomers (ASOs) were exogenously delivered to bind and silence the corresponding transcripts^113^. This allowed targeting of phage genes without needing genetic tractability, which is often a limitation of clinical isolates. ASOs were used to discover 56 phage genes whose knockdown impacted phage propagation^113^.

Lastly, phage transposon sequencing platforms have been developed that enable systematic identification of essential and non-essential genes in lytic phages^152,153,154^. This approach allows conditional genetic screens under diverse environments and/or host strains for functional genomics of jumbo phages. Using phiKZ^152^ and PCH45^153^ as models, the technique revealed ~110 out of 371 genes and ~102 out of 225 genes, respectively, that confer fitness to the phage under tested conditions. The considerable fraction of non-essential genes (~55–70%) indicates that jumbo phage genomes carry substantial accessory genes that, however, could be conditionally essential.

### Biotechnological and therapeutic potential

Phages are regaining attention as self-amplifying therapeutic agents, particularly against antibiotic-resistant pathogens. Compared to smaller phages, jumbo phages offer several translational advantages. Jumbo phages with a host range typically broader than that of smaller phages are promising candidates for phage therapy. For example, jumbo phages found on *Enterobacter* were also found capable of creating plaques on pathogenic *Escherichia, Klebsiella, Serratia, Salmonella, Shigella, Providencia, Citrobacter* and *Cronobacter*^24^. Moreover, since jumbo phages encode extensive replication, transcription, and metabolism machinery, their infection cycle is considered less dependent on host factors compared to that of the smaller phages. Lastly, jumbo phages are often more resistant to DNA-targeting bacterial immune systems, including restriction-modification and CRISPR-Cas, via the nucleus and the EPI vesicle, as well as diverse anti-defense modules that lower the risk of host resistance development.

An increasing number of jumbo phages have been isolated and characterized for their lytic potential against biofilm-forming and drug-resistant *P. aeruginosa* strains^155^. Notably, the jumbo phage PA5oct is effective in reducing *P. aeruginosa* loads in airway infection models^156^, while various jumbo phages have potent activity against clinical isolates and biofilms^53,157,158^. Moreover, some jumbo phages have the added benefit of inducing collateral susceptibility in pathogens to antibiotics. For example, resistance to *P. aeruginosa* phage OMKO1 results in increased antibiotic sensitivity, because mutation of the phage receptor, a porin, hampers antibiotic efflux^159^. Additionally, combined phage-antibiotic treatments in murine pneumonia models show greater bacterial reduction than either treatment alone^121,160^. On the other hand, various antibiotics were shown to strongly block phiKZ replication in a dose-dependent manner, highlighting the importance of systematically assessing the synergistic or antagonistic effects between jumbo phages and antibiotics^107^.

However, jumbo phages also raise distinct challenges relative to smaller phages. Their large virions are more readily cleared in vivo and may show altered pharmacokinetics and tissue distribution, with limited data so far from animal models^121^. The vast number of hypothetical genes complicates safety assessment. Broad host range, while therapeutically convenient, may increase off-target bacterial killing compared with more host-restricted conventional phages.

Alternative to applying live jumbo phages, purified phage-derived enzymes such as endolysins from phiKZ and phage EL show antibacterial activity, especially when combined with membrane-permeabilizing agents^161–163^. In food safety, jumbo phage CR5 eradicated *Cronobacter sakazakii* from infant formula^58^, and cocktails of jumbo phages have been effective against *Aeromonas salmonicida* in aquaculture^164^ and *Vibrio parahaemolyticus* in shrimp^97^. For sterilization purposes, jumbo phages successfully eliminated drug-resistant *P. aeruginosa*-contaminated eye drops^165^. Finally, as jumbo phages are capable of general transduction and packaging of large amounts of DNA in their heads, they may represent possibilities for gene delivery analogous to T4-based systems^166–169^.

Phage biology has long supplied tools for biotechnology, including λ Red recombination, T4 ligase, T7 RNA polymerase, and M13 phage display. Jumbo phages, with their large genomes and as yet uncharacterized proteins, offer a promising source of future discoveries.

### Conclusion and outlook

Jumbo phages, with their large genomes, giant virion sizes, and intricate lifecycles, are redefining long-held assumptions about bacteriophage complexity. Despite major advances in recent years, our understanding of jumbo phage biology remains limited, with many fundamental questions still unanswered. Are there additional, yet undiscovered, viral organelles encoded by jumbo phages? How many eukaryotic-like characteristics of jumbo phages might still be uncovered? What evolutionary pressures shape the genomic architecture and complexity of jumbo phages? How does the viral genome transit from the EPI vesicle into the phage nucleus? How are nucleotides, mRNAs, and proteins selectively imported and exported across the EPI vesicle and the nucleus shell? How do jumbo phages evade or suppress diverse host immune systems? What is the function of the small, uncharacterized genes present in many jumbo phage genomes? Are there undiscovered classes of MGEs encoded within, or parasitizing, jumbo phages? What is the therapeutic potential of jumbo phages in overcoming multidrug-resistant infections? To what extent will structural prediction tools (e.g., AlphaFold, Foldseek) and metagenomic mining reveal unknown jumbo phage functions? Such questions may guide future research priorities in exploring the jumbo phage jungle.
